# Best- and Worst-Case Scenarios for the Douro Winemaking Region: Dynamic Crop Modelling and Ensemble Projections for Yield, Alcohol Content, and Phenology

**DOI:** 10.3390/plants14162466

**Published:** 2025-08-08

**Authors:** Helder Fraga, Emanuele Serra, Nathalie Guimarães, Nazaret Crespo, António Fernandes, Christoph Menz, João A. Santos

**Affiliations:** 1 Centre for the Research and Technology of Agro-Environmental and Biological Sciences (CITAB), Institute for Innovation, Capacity Building, and Sustainability of Agri-Food Production (Inov4Agro), University of Trás-os-Montes e Alto Douro (UTAD), P.O. Box 1013, 5000-801 Vila Real, Portugal; nsguimaraes@utad.pt (N.G.); nazaret@utad.pt (N.C.); acpf91@utad.pt (A.F.); jsantos@utad.pt (J.A.S.); 2 Department of Agricultural Sciences, University of Sassari, Viale Italia 39A, 07100 Sassari, Italy; emanuele.serra@iusspavia.it; 3 University School for Advanced Studies IUSS Pavia, Palazzo del Broletto, Piazza della Vittoria 15, 27100 Pavia, Italy; 4 Euro-Mediterranean Centre on Climate Change (CMCC) Foundation, Impacts on Agriculture, Forests and Ecosystem Services (IAFES) Division, Via De Nicola 9, 07100 Sassari, Italy; 5 Potsdam Institute for Climate Impact Research e. V., PIK, Telegrafenberg A 31, 14473 Potsdam, Germany; christoph.menz@pik-potsdam.de

**Keywords:** viticulture, climate change, future scenarios, wine, grapevine, STICS, multi-model

## Abstract

Climate change is expected to significantly reshape viticulture across traditional wine regions, including the Douro winemaking region (DWR) in northern Portugal. This study evaluates projected impacts of climate change on key viticultural parameters, such as grapevine yield, phenology, and potential alcohol content, using an ensemble of high-resolution downscaled climate simulations for the recent-past (1986 to 2015) and for two emission scenarios: SSP1–2.6 (low-emissions pathway) and SSP5–8.5 (high-emissions pathway), for mid-century (2041–2070). Spatial and temporal analyses reveal a consistent and robust signal of change across all indicators, with magnitude and variability increasing under SSP5–8.5. Yield projections indicate a widespread decline across the region (−1 to −3 t/ha), especially under SSP5–8.5, with particularly strong reductions in currently high-yielding areas, such as Douro-Superior. This spatial heterogeneity suggests heightened vulnerability throughout the DWR, underscoring the importance of targeted adaptation strategies. Phenological analysis shows a marked advancement in flowering dates, shifting by up to 30 days earlier in the season, amplified under SSP5–8.5. These changes could impact grape development, increase exposure to early-season frost events, and disrupt traditional vineyard management schedules. Furthermore, potential alcohol content is projected to rise substantially across the region, with increases exceeding 2% vol in some areas under the more severe scenario. This trend may challenge wine typicity, regulatory classifications and geographical boundaries of the denominations of origin, and quality control, requiring both vineyard and oenological adaptations to manage elevated sugar levels. These findings point to significant, spatially variable climate-driven transformations in Douro viticulture. While some impacts may be partially mitigated under SSP1–2.6, SSP5–8.5 may require urgent adaptation to preserve wine quality, socioeconomic sustainability, and regional identity.

## 1. Introduction

Viticulture is a major global sector, with approximately 8 million hectares of vineyards worldwide dedicated to grape cultivation in 2022 [1]. Global wine production reached 260 million hectolitres in the same year, while wine consumption stood at 232 million hectolitres [1]. In the European Union (EU), home to some of the most famous wine-producing regions, nearly 3 million jobs and a €130 billion annual contribution to GDP are supported by the wine industry [2]. Meanwhile, in the United States of America (USA), the wine sector supports around 1.75 million jobs, contributes over $102 billion in wages, and generates $323 billion [3]. These numbers highlight the vast economic, cultural, and social significance, shaping rural economies, sustaining regional identities, and driving international trade. Italy leads with over 47 million hectolitres, followed closely by France and Spain. Outside Europe, the USA ranks as the fourth-largest producer, while Argentina, Australia, Chile, and South Africa also play significant roles in the global wine landscape. Portugal is also among the top ten wine-producing countries, with a production of approximately 7 million hectolitres annually.

**Figure 1:**
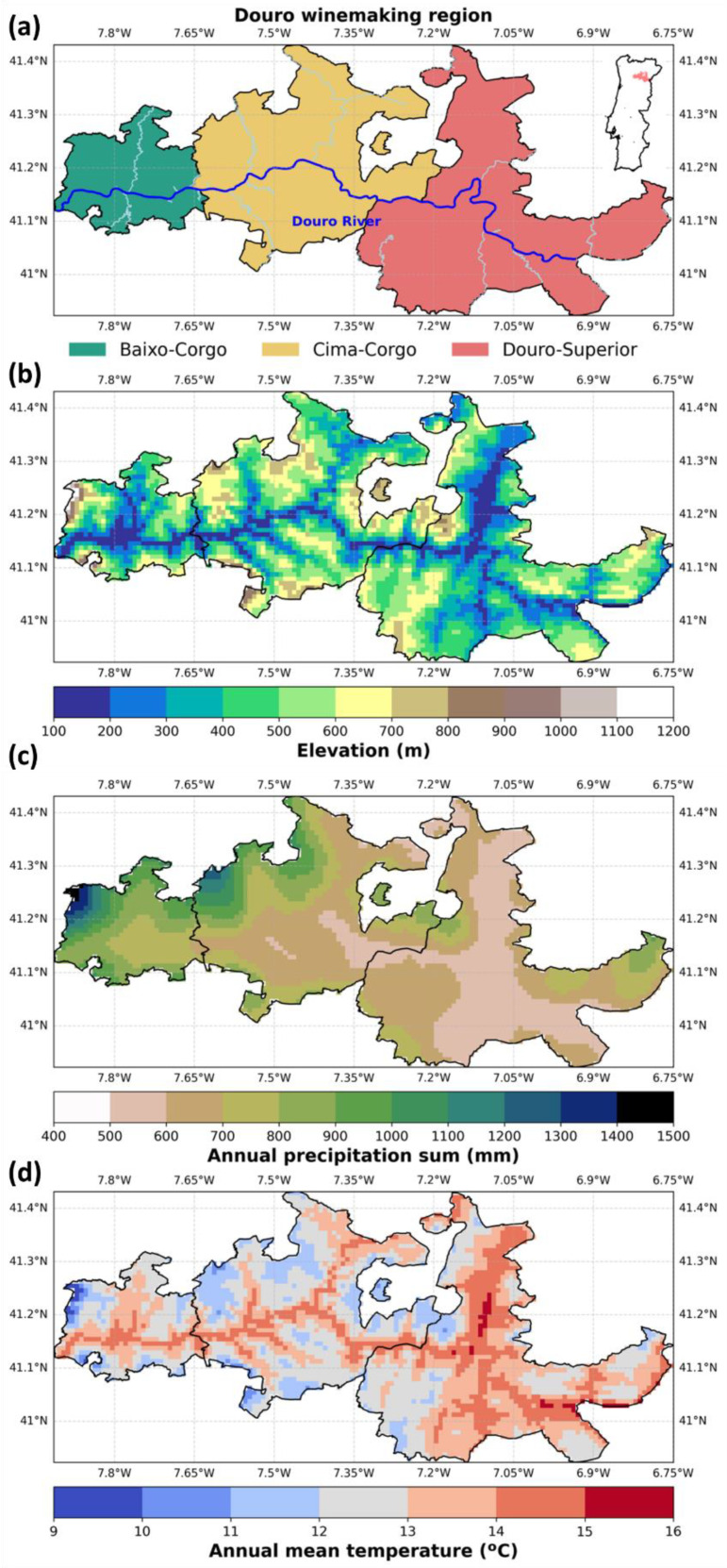
(**a**) Geographical representation of the DWR along with the three subregions. Main water bodies are represented by a blueish line. (**b**) Elevation (m), (**c**) annual precipitation sum (mm), and (**d**) annual mean temperature (°C) in the DWR, for 1986–2015.

The Douro wine region (DWR), located in northern Portugal along the Douro River, is one of the oldest demarcated wine regions in the world, officially established in 1756, and currently has two denominations of origin: Douro and Porto. In 2024, the DWR produced around 1.6 million hl of wine, originating from ~43,000 ha of vineyards, which generated an income of ~624 million € [4]. This World Heritage region for UNESCO is widely renowned for its production of fortified Port wines. The region has a rich viticultural tradition, spanning several centuries, and is divided into three subregions (Figure 1a), namely Baixo-Corgo, Cima-Corgo, and Douro-Superior, each characterized by distinct characteristics, or terroir, such as soil composition and climate. These subregions contribute to the complexity and diversity of the wines produced, with Cima-Corgo (central subregion) being currently considered the most prestigious, due to its favourable climatic conditions and superior grape quality [5]. Over time, the DWR has expanded its production to include high-quality dry table wines, reflecting evolving viticultural practices and market demands [6]. The DWR is one of the most important mountain viticulture areas in the word, due to its rugged orography and the steep slopes (Figure 1b).

The DWR produces a diverse range of wines, predominantly categorized into fortified Port wines and dry table wines [7]. Port wine, traditionally sweet and fortified with grape spirit, remains a major product with a strong global market presence. The evolution of wine styles in the DWR reflects a balance between heritage and innovation, adapting to global wine market dynamics, and also boosting the DWR oeno-tourism [8]. In recent decades, there has been a marked increase in the production of non-fortified, dry red and white wines, driven by both consumer preferences and advancements in viticulture and oenology [4]. This diversification aligns with broader trends toward sustainability, organic practices, and appellation-specific quality improvements. Additionally, there is a large set of indigenous grape varieties, which contribute to unique sensory profiles and regional identity, also known as wine typicity [9].

It is well known that climate significantly influences vine phenology, grape composition, and ultimately wine quality [10,11]. As an example, temperature is the leading determinant for grapevine phenology, while precipitation levels significantly influence soil moisture availability, which is a critical determinant of vine water stress, which in turn affects yield and quality attributes. As such, climate change poses significant challenges to viticulture in the DWR, primarily through increased temperatures, altered precipitation patterns, and greater frequency of extreme weather events [12,13]. Rising temperatures can accelerate grape ripening and advance harvest times, leading to higher sugar accumulation, potentially elevated alcohol content, and lower acidity in wines, which may disrupt traditional flavour profiles. Extreme temperatures can lead to maturation stoppages and yield losses [14]. Shifts in precipitation and increased drought risk may exacerbate vine water stress, affecting yield and grape composition [10,11]. Additionally, phenological phases such as budburst, flowering, and veraison may advance, altering the synchrony with favourable climatic conditions. These changes threaten the region’s viticultural sustainability, necessitating adaptive management strategies to preserve wine quality and production stability.

Dynamic crop models, such as STICS (Simulateur mulTIdisciplinaire pour les Cultures Standard), are valuable tools for assessing the impacts of environmental variability and management practices on crop growth and yield [15]. STICS integrates physiological, climatic, and soil parameters to simulate crop development stages, biomass accumulation, and yield under varying scenarios [16]. In viticulture, such models enable the prediction of phenological timings, water requirements, and potential grape yield under current and future climate conditions [17,18,19]. By incorporating detailed climate data and soil characteristics, STICS facilitates the assessment of adaptive strategies, including irrigation scheduling and cultivar selection, thus supporting decision-making processes, aiming to mitigate climate change impacts on grape production and quality attributes [19].

Several studies have investigated these issues across various wine-producing regions, such as the Bordeaux region in France [20], the Napa Valley in the United States [21], and the Barossa in Australia [22]. However, most of these studies still rely on the former Representative Concentration Pathways (RCPs) [23]. Furthermore, these approaches usually rely on climate data at relatively coarse spatial resolutions (typically 12.5 km), which may not adequately capture the complex topography and intricate microclimates characteristic of regions like the DWR, where sharp gradients in elevation, slope, and solar exposure strongly influence vine development and grape quality. In contrast, our study leverages a very high-resolution, state-of-the-art ensemble of climate models based on the latest Shared Socioeconomic Pathways (SSPs) [24,25], allowing for more precise simulations of local climate variability and its impact on viticulture. This enhanced resolution and updated scenario framework provide a more robust basis for assessing the impact of climate change on Douro vineyards.

**Figure 2:**
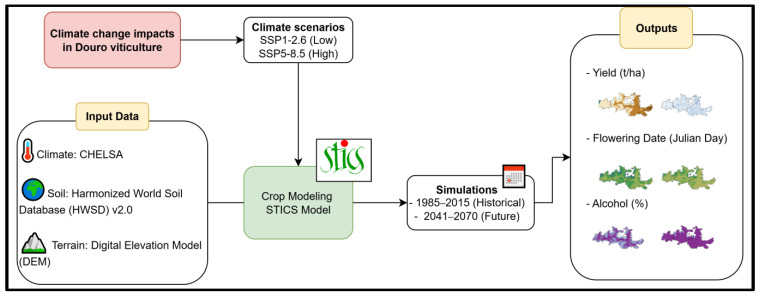
Graphical representation of the modelling approach in the current study.

The current study applies a comprehensive ensemble of state-of-the-art climate models, coupled with the STICS dynamic crop model, to assess the future impacts of climate change on three key factors influencing viticulture in the Douro region: yield, alcohol content, and phenological development (Figure 2). By integrating high-resolution climate projections with detailed physiological crop responses, this study provides robust, region-specific projections of grapevine performance. It considers two contrasting socio-economic climate scenarios, SSP1-2.6 and SSP5-8.5, which together represent a wide range of plausible future conditions. This scenario-based approach enables a detailed evaluation of how changes in temperature, precipitation, and extreme weather events may affect grape development and wine quality. The novelty of the study lies in its coupling of fine-scale climate modelling with a crop-specific simulation framework applied to a globally recognized wine region. The results are intended to inform adaptive management strategies and support sustainable policy-making to enhance the resilience of Douro wine production under climate change. The objectives of this study are as follows: (1) to quantify the potential effects of future climate change on grapevine yield, phenology, and grape composition in the Douro region; (2) to assess how these impacts may differ under low- and high-emissions scenarios; and (3) to provide model-based insights that support adaptive management strategies and sustainable policy-making aimed at ensuring the long-term resilience of Douro wine production.

## 2. Results

### 2.1. Yield Projections

**Figure 3:**
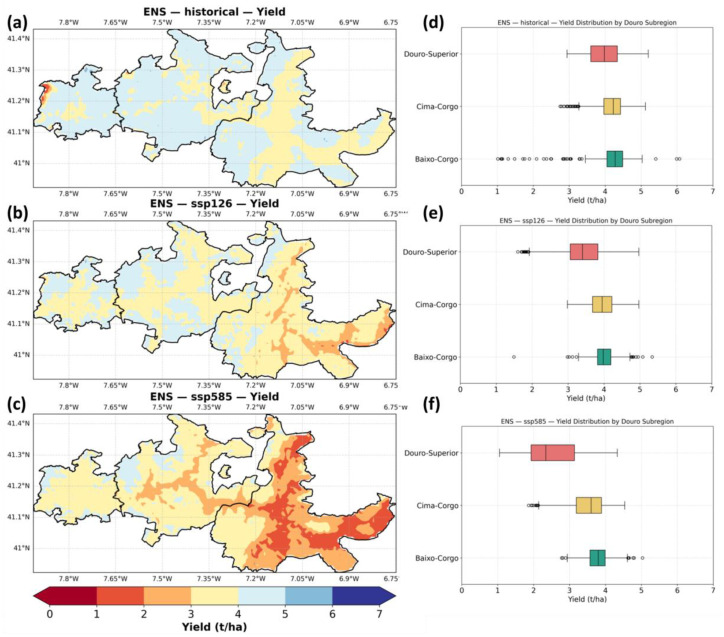
Spatial representation of simulated grapevine yield (t/ha) for the (**a**) historical, (**b**) SSP1–2.6, and (**c**) SSP5–8.5 scenarios. Box-plots (t/ha) of the regional yield for the three subregions (Baixo-Corgo, Cima-Corgo, and Douro-Superior), for the (**d**) historical, (**e**) SSP1–2.6, and (**f**) SSP5–8.5 scenarios (outliers are also represented as circles). For succinctness, SSP1–2.6 is shown to as ssp126, and SSP5–8.5 as ssp585.

Figure 3a–c show the spatial distribution of ensemble median simulated yield for the historical period (1986–2015) and two future scenarios, SSP1–2.6 and SSP5–8.5, for the period 2041–2070. Under historical conditions (Figure 3a), yields are relatively homogeneous across the region, with slightly lower values in the eastern areas, particularly within Douro Superior. Under SSP1–2.6 (Figure 3b), a yield reduction is apparent, especially in Douro-Superior and parts of Cima-Corgo. The SSP5–8.5 scenario (Figure 3c) shows a pronounced decline in yield across the entire region, with the strongest reductions concentrated in the eastern and southern parts of Douro-Superior, reflecting the effects of increased heat and water stress under the high-emissions pathway.

Figure 3d–f present the distribution of simulated yields for each of the three Douro subregions. Historically, Baixo-Corgo exhibits the highest median yield, followed by Cima-Corgo and Douro-Superior. This regional pattern persists under SSP1–2.6 but with slightly reduced medians. Under SSP5–8.5, all subregions experience a marked decline in yield, with Douro-Superior showing the largest increase in interquartile range, suggesting both a drop in productivity and increased spatial variability. Outliers are more frequent under historical and SSP1–2.6 conditions, indicating greater local heterogeneity in yield, whereas under SSP5–8.5, yields become more uniformly suppressed across the regions.

**Figure 4:**
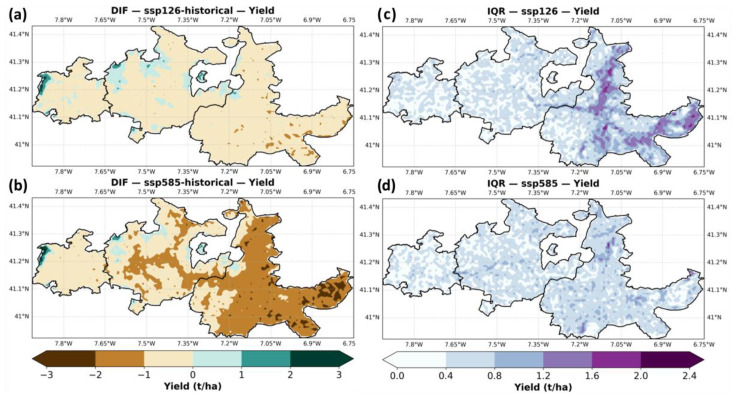
Difference in yields between historical and (**a**) SSP1–2.6 and (**b**) SSP5–8.5 (future minus historical). Grapevine yield (t/ha) interquartile range (percentile 75 minus 25) between the ensemble of climate models, for (**c**) SSP1–2.6 and (**d**) SSP5–8.5. For succinctness, SSP1–2.6 is shown to as ssp126, and SSP5–8.5 as ssp585.

Figure 4 illustrates the projected differences in grapevine yield (t/ha) between the historical baseline (1986–2015) and two future climate scenarios (2041–2070), SSP1–2.6 and SSP5–8.5, as well as the spatial distribution of inter-model uncertainty represented by the interquartile range (IQR). Panels (a) and (b) show the difference in ensemble median yield under SSP1–2.6 and SSP5–8.5, respectively. Under SSP1–2.6 (panel a), yield changes are relatively modest, with much of the region showing small decreases (−1 to −2 t/ha), especially in the eastern parts of Douro-Superior. Localized areas in Baixo-Corgo show slight yield increases, mostly in high-altitude zones (Alvão and Marão mountains). In contrast, under SSP5–8.5 (panel b), significant yield reductions dominate the region, with widespread losses exceeding −2 t/ha, particularly across the Douro-Superior and parts of Cima-Corgo. Minor positive anomalies persist only in limited high-altitude zones.

Panels (c) and (d) display the interquartile range of ensemble yield projections under SSP1–2.6 and SSP5–8.5, respectively. Under SSP1–2.6 (panel c), model spread remains moderate, with IQR values mostly below 1 t/ha but with some localized uncertainty hotspots (1.5–2.4 t/ha) along eastern slopes of Douro Superior. Under SSP5–8.5 (panel d), ensemble uncertainty becomes more spatially widespread but remains within similar magnitudes, suggesting consistent model agreement on the direction of change, particularly in areas with the greatest projected losses.

### 2.2. Flowering Date Projections

**Figure 5:**
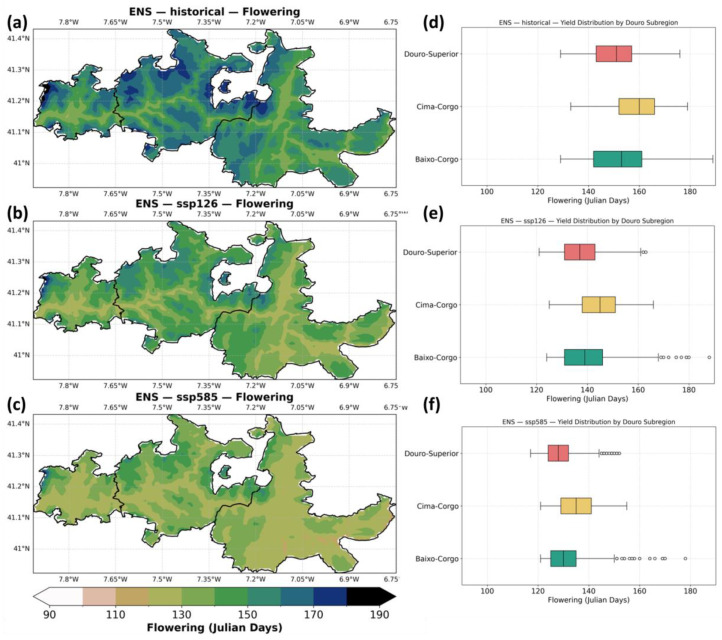
Same as Figure 3, but for flowering dates (Julian days).

Figure 5 shows the ensemble median projections for grapevine flowering dates. In the historical period (panel a), flowering generally occurs from mid-May to early June (Julian days 140–160), with earlier flowering in the warmer eastern areas of Douro-Superior and later flowering in the cooler and moister Baixo-Corgo. Under SSP1–2.6 (panel b), flowering is projected to advance by approximately one to two weeks, occurring mostly between early and late May (Julian days 130–150). The shift is stronger under SSP5–8.5 (panel c), where flowering occurs as early as mid-April to mid-May (Julian days 120–140), especially near the river valleys (low elevation zones). Figure 5d–f summarize these changes by subregion. In the baseline (panel d), median flowering occurs in late May in Douro-Superior, slightly later in Cima-Corgo, and around early June in Baixo-Corgo. Under SSP1–2.6 (panel e), all three subregions show earlier flowering by about one week. Under SSP5–8.5 (panel f), flowering advances by up to two weeks, reaching mid-May in Douro Superior, shifting to mid-to-late May in Cima Corgo, and to late May in Baixo Corgo.

**Figure 6:**
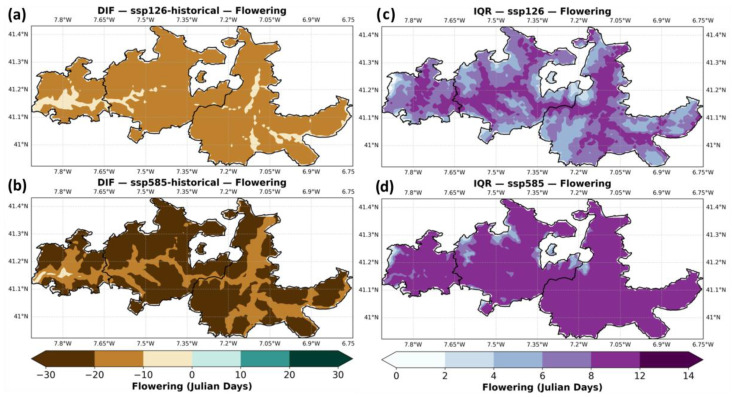
Same as Figure 4, but for flowering dates (Julian days).

Figure 6 shows the difference between future scenarios and the historical baseline, along with the ensemble uncertainty (IQR). Differences between scenarios are particularly significant, while in SSP1–2.6 the advance of flowering may reach up to -20 days, in SSP5–8.5 the advance will reach up to one month. Regarding the model agreement, in SSP5–8.5, IQR tends to be relatively high in all areas (up to 12 days), whereas, in SSP1–2.6, the areas closer to the river, tend to show higher values of ensemble uncertainty, with the rest of Douro showing low IQR values (<4 days).

### 2.3. Alcohol Content Projections

**Figure 7:**
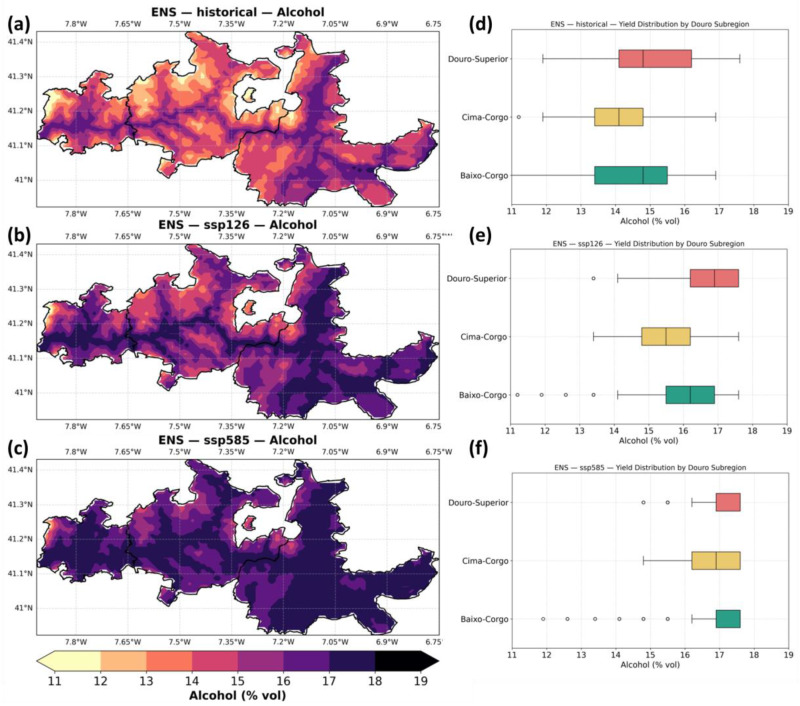
Same as Figure 3, but for alcohol content (% vol).

Figure 7 shows the ensemble median of predicted alcohol content (% vol) in grape must across the Douro region, for both the baseline and future scenarios (SSP1–2.6 and SSP5–8.5). In the baseline (panel a), alcohol content ranges between 13% and 16.5%, with higher values generally occurring in the warmer eastern areas of Douro-Superior, and lower values in the cooler, Atlantic-influenced Baixo-Corgo. Under SSP1–2.6 (panel b), alcohol content increases moderately, with much of Douro-Superior and Cima-Corgo exceeding 16%, and Baixo-Corgo rising above 14%. This trend intensifies under SSP5–8.5 (panel c), with most of the region showing alcohol contents between 16% and 17.5%, particularly across Douro-Superior, and even the western Baixo-Corgo surpassing 15% in many areas. It should be noted that STICS is limited to 17.5% vol as a maximum (cut-off value).

The subregional boxplots (panels d–f) confirm these spatial patterns. Historically (panel d), median alcohol content is around 15.5% in Douro-Superior, 14.5% in Cima-Corgo, and just under 14% in Baixo-Corgo. Future projections under SSP1–2.6 (panel e) indicate increases of approximately 1% across all subregions, with Douro-Superior reaching medians near 16.5%. Under SSP5–8.5 (panel f), alcohol content increases further, with medians exceeding 17% in Douro-Superior and reaching 15–16% in Baixo-Corgo.

**Figure 8:**
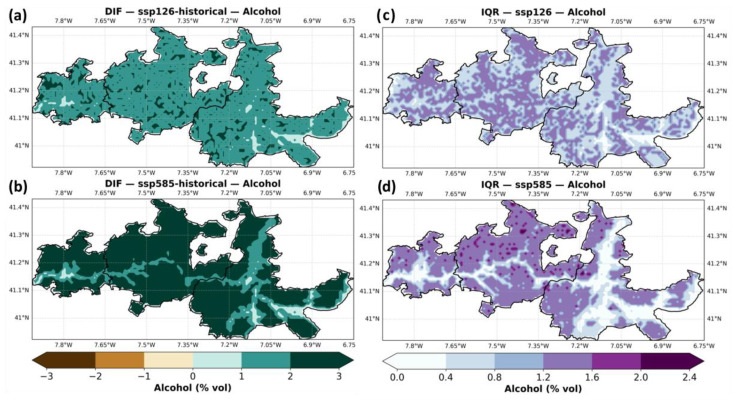
Same as Figure 4, but for alcohol content (% vol).

Figure 8a summarizes the aforementioned changes. Under SSP1–2.6 (panel a), alcohol content is projected to increase moderately across most of the Douro region, with widespread gains between +0.5% and +1.5% vol. In SSP5–8.5 (panel b), these increases become more pronounced, especially at higher elevations in the south-eastern areas, where changes often exceed +2% vol. The largest alcohol gains under SSP5–8.5 occur in Douro-Superior, but meaningful increases are evident even in Baixo-Corgo.

The ensemble uncertainty (IQR), assessed by the interquartile range (panels d,e), shows variability across the individual models. Under SSP1–2.6 (panel c), uncertainty is moderately high (IQR ~0.6–1.4% vol), particularly along central and eastern subregions. Under SSP5–8.5 (panel d), the IQR increases slightly, especially in northern Cima-Corgo and parts of Baixo-Corgo, with values reaching up to 2.0% vol in some areas, indicating higher climate-model-driven uncertainty under stronger forcing.

## 3. Discussion

The present study assesses the potential impacts of climate change on viticulture in the Douro Wine Region by analysing crop model simulations driven by future projections. The modelling setup includes two future scenarios, SSP1–2.6 representing a low-emissions pathway and SSP5–8.5 reflecting a high-emissions trajectory, capturing the plausible range of future climatic conditions and associated impacts, while the use of a multi-model ensemble enables a robust assessment of uncertainty, which is essential for interpreting regional-scale climate projections. Furthermore, the STICS soil–crop model was used in this assessment, being widely recognised for its ability to simulate grapevine responses under varying environmental conditions, with a high reliability in viticultural applications [26,27,28,29,30].

The results underscore the complex impacts of climate change on viticulture in the Douro Valley, with significant and consistent signals emerging across key metrics of grapevine performance, in yield, phenology, and alcohol content. These changes are projected to intensify under stronger warming scenarios, particularly under SSP5–8.5, posing both challenges and opportunities for vineyard management, regional wine typicity, and long-term sustainability.

Grapevine yield projections suggest that climate change is likely to negatively affect productivity across the region, with the severity and spatial extent of impacts increasing markedly under the high-emission scenario SSP5–8.5. While some inter-model variability exists, the ensemble median reveals a strong viticultural suitability decline, particularly in Douro-Superior. This highlights the vulnerability of areas currently contributing substantially to production, where losses may not only challenge economic viability but also exacerbate inequalities in regional exposure, vulnerability and resilience to climate stressors. The spatial heterogeneity observed in yield responses underscores the importance of fine-scale assessments to guide targeted ad hoc adaptation measures.

The analysis of flowering phenology reveals a consistent and robust signal of earlier onset across the Douro, driven by rising temperatures. Under both SSP1–2.6 and SSP5–8.5, flowering advances by several weeks, with the shift becoming more pronounced under higher forcing. These phenological changes carry significant implications for vineyard management, including adjustments to pruning, canopy control, or pest/disease risk mitigation. Earlier flowering also raises concerns about increased exposure to late spring frost events and potential mismatches between phenological stages and optimal climatic windows for fruit set and ripening. Consequently, long-term planning will need to account for these dynamics to maintain grape quality and harvest efficiency.

In terms of alcohol content, the results indicate a widespread and consistent increase across the region, with projections suggesting a rise in potential alcohol levels, particularly under SSP5–8.5. This trend is accompanied by increased uncertainty in ensemble projections, especially in northern and coastal subregions. The implications of this shift are far-reaching, potentially altering wine style, balance, and market classification. Managing sugar accumulation and controlling alcohol content may become a central adaptation challenge, requiring viticultural interventions (e.g., canopy management, irrigation, varietal selection) and oenological adjustments during winemaking. Given the cultural and regulatory importance of wine typicity in the Douro, these compositional changes could challenge traditional production regulations and appellation criteria.

When compared with findings from other viticultural regions, the projected impacts of climate change on the Douro Valley align closely with broader global trends observed in grapevine responses to warming temperatures [31]. For instance, studies in Mediterranean regions, such as La Rioja (Spain) [32], Tuscany [33,34] and Trento (Italy) [35], Franconia (Germany) [36], and several regions in France [37], have reported similar advances in phenological stages, including earlier budburst, flowering and veraison, consistent with our observations. Like the Douro, these regions also face challenges related to yield declines under high-emission scenarios, recognizing the vulnerability of traditional wine-producing areas to climate-induced stress. Moreover, the magnitude of phenological shifts reported in these studies is comparable with flowering advancing by two to four weeks under high warming scenarios, underscoring the widespread nature of this emerging trend.

Several studies have also documented increases in grape sugar content and potential alcohol levels associated with rising temperatures, mirroring our projections for the Douro Valley. For example, van Leeuwen and Destrac-Irvine [38] demonstrated significant modification in grapevine composition under future climate scenarios, raising concerns about alterations in wine style and balance similar to those highlighted in our analysis. This trend toward higher alcohol content is increasingly viewed as a global viticultural challenge, requiring adaptive strategies, such as modified canopy management or the introduction of lower sugar-accumulating grape varieties, as suggested by [39].

In contrast with the results found for the DWR, some studies are reporting potential yield gains in cooler, higher-altitude regions due to warming, such as some areas of southern England [40]. Furthermore, new warming conditions are becoming more frequent in recent-past cool climate areas, which may lead to new types of wine [41]. Conversely, in the warm climate of the DWR, results emphasise a net decline in yield, particularly in the warmer and drier Douro-Superior sub-region. This is attributed to the stronger warming and drying trends, together with the soil-water limitations, which are characteristic of the upper Douro valley, thus exacerbating heat stress and drought impacts on grapevines. Such heterogeneity echoes findings from recent assessments in California’s wine regions, where drought and heat stress have led to contrasting yield outcomes depending on local conditions and water availability [42,43,44]. These comparisons highlight the necessity of region-specific adaptation planning.

The increased uncertainty in ensemble projections observed in northern and coastal subregions of the Douro reflects a pattern seen in other coastal viticultural areas, such as the Willamette Valley in Oregon [45,46]. Coastal microclimates introduce complexities in climate modelling due to maritime influences and fog patterns, which can modulate phenological and compositional responses. This underlines the importance of incorporating fine-scale climatic and topographic data in future modelling efforts to better capture local variability and inform targeted vineyard management.

Uncertainty is an inherent aspect of climate change impact assessments, arising from multiple sources, including greenhouse gas emission trajectories, climate model structures, and the responses of crop models to environmental variables. In this study, an ensemble approach was adopted, incorporating two contrasting socio-economic scenarios (SSP1-2.6 and SSP5-8.5) and outputs from four distinct global climate models (GCMs). This framework captures a broad range of possible future climatic conditions, helping to bracket the range of uncertainty associated with emissions pathways and inter-model variability. While ensemble statistics such as the mean and spread provide an initial understanding of this variability, further exploration of uncertainty is acknowledged as an important area for future work. Nonetheless, the current ensemble design provides a structured and informative basis for evaluating potential impacts on Douro viticulture under plausible future conditions.

Regarding the crop modelling approach, some limitations of the present study should also be acknowledged. While the STICS model performed well in simulating key viticultural parameters, its assumptions introduce important limitations. The use of a single cultivar or homogeneous varietal input may not capture the variability commonly found within DWR vineyards. Likewise, the assumption of uniform management practices, such as canopy management, oversimplifies the diverse strategies used by growers that often vary by site, season, and production objectives. These simplifications limit its applicability under heterogeneous field conditions and should be carefully considered when interpreting results or using the model to support vineyard decisions.

Nonetheless, the STICS model remains an essential tool for detecting and analysing climate change signals in viticulture by simulating long-term trends in phenology, yield, and alcohol content under future climate scenarios. The findings of this study highlight the need for timely and carefully designed adaptation measures. These may include adjusting planting dates, selecting heat- and drought-tolerant grapevine varieties, modifying canopy management practices to reduce heat stress, implementing precision irrigation strategies, and shifting vineyard locations to cooler microclimates or higher altitudes where feasible. By delivering precise forecasts and comprehensive scenario analyses, the model empowers growers and stakeholders to make informed decisions that strengthen vineyard resilience, preserve grape quality, and ensure economic sustainability amid evolving climatic conditions. Ultimately, these adaptation strategies contribute to sustainable viticulture, mitigate risks associated with climate variability, and support the long-term stability and competitiveness of the wine industry.

## 4. Material and Methods

### 4.1. Study Area

The DWR exhibits Mediterranean-type climates, generally characterized by hot–dry summers and mild–wet winters [47]. However, the region’s climatic heterogeneity across subregions fosters diverse microclimates that support the cultivation of various grape varieties [48]. The three sub-regions exhibit climatic differences largely attributable to their longitudinal position and varying degrees of Atlantic Ocean influence. Baixo-Corgo, the westernmost subregion, closest to the North Atlantic Ocean, experiences the most maritime conditions, with relatively cooler temperatures and higher annual precipitation, ranging from 700 to 1200 mm [49] (Figure 1c,d). This maritime influence moderates summer heat and reduces the risk of drought stress, fostering more consistent vine growth. Cima-Corgo, situated eastward and further inland, presents a transitional climate, between maritime and continental, characterised by warmer temperatures and lower precipitation (approximately 700 to 900 mm annually), as the maritime influence weakens towards inner Iberia. Douro-Superior, the easternmost and most inland subregion, exhibits higher continentality, with the highest summer temperatures and lowest precipitation levels, often below 600 mm annually, reflecting minimal Atlantic influence. This climatic gradient significantly impacts vine phenology, water availability, and grape quality, contributing to the distinct terroirs and wine profiles characteristic of each subregion [50]. Average annual temperatures also show this longitudinal pattern, ranging from approximately 9 °C to 17 °C, with the Douro-Superior showing warmer conditions overall.

According to the Portuguese Vine & Wine Institute (IVV), the DWR comprises approximately 43,000 hectares of vineyards, distributed across Baixo-Corgo (~14,000 ha), Cima-Corgo (~20,000 ha), and Douro-Superior (~9000 ha) [51]. The maximum yield allowed by law is 55 hl/ha (about 7.5 t/ha), while the average productivity typically ranges from 4 to 6 t/ha (IVDP). The main varieties by area are Touriga-Franca (25%), Tinta-Roriz (14%, syn: Tempranillo), and Touriga-Nacional (10%) [4]. Flowering tends to occur between late May and early June (Julian days 140–160), and wine production dominated by red wines (~80%), followed by white (~15%) and rosé (~5%), with average alcohol content between 12.5% and 14.5% by volume.

### 4.2. Crop Model Description

The STICS (Simulateur mulTIdisciplinaire pour les Cultures Standard) crop model is a process-based, dynamic simulation model that integrates key biophysical processes governing crop development, growth, and yield under varying environmental and management conditions [52]. It simulates crop phenology, biomass accumulation, water and nitrogen balances, and final yield, based on daily meteorological inputs, soil characteristics, crop-specific parameters, and management practices. Originally developed for arable crops, STICS has been successfully adapted and applied to perennial crops, including grapevine, to assess the impacts of climate variability and management practices on crop performance. Previous studies have demonstrated the model’s applicability to viticulture in Mediterranean climates, including in the Douro region, where it has been used to simulate phenological responses and productivity under climate change scenarios [53]. Model runs and parameterisations are described below.

### 4.3. Climate Model Data

Very high-resolution climate data were generated through a hybrid statistical–dynamical downscaling of four General Circulation Models (GCMs) to better resolve the complex topography of the Douro region. The CHELSA (Climatologies at High Resolution for the Earth’s Land Surface Areas) downscaling approach was employed for this purpose [24]. The high-resolution downscaling technique integrates orographic effects, wind fields, and boundary layer processes to improve the spatial accuracy of climate variables in mountainous and topographically diverse regions. As input for the downscaling 4 GCMs (Table 1) from the Inter-Sectoral Impact Model Intercomparison Project (ISIMIP3b) [54,55], were selected. These models are already bias-adjusted and downscaled versions of model simulations from the Coupled Model Intercomparison Project Phase 6 (CMIP6) [54,55]. The output data was downscaled to a spatial resolution of 30 arc-seconds (~1 km), providing daily time series for key variables required by the STICS model. These included daily maximum temperature (Tmax), minimum temperature (Tmin), and surface shortwave radiation (Rsds), ensuring a detailed and spatially explicit climatic input dataset for the simulations. Daily data were obtained for the historical period (HIST) from 1986 to 2015 and for the future period 2041 to 2070 under two contrasting anthropogenic radiative forcing scenarios: SSP1–2.6 (low-emissions, sustainability-focused pathway) and SSP5–8.5 (high-emissions, fossil-fuel-intensive pathway). These SSPs represent a wide range of possible socio-economic development pathways and are used as baseline scenarios for the latest assessment report of the Intergovernmental Panel on Climate Change [56]. This dual-scenario approach enables the assessment of both “optimistic” and “pessimistic” projections, allowing for a comprehensive evaluation of climate change impacts on viticulture in the Douro region under varying socio-economic and greenhouse gas concentration trajectories.

### 4.4. Soil and Terrain Data

Soil data were obtained from the Harmonized World Soil Database (HWSD) v2.0 [61], which provides globally consistent layers for key pedological parameters. All parameters are shown in Table 2, such as soil depth, carbon-to-nitrogen (C/N) ratio, soil texture (percentage of sand, silt, and clay), and classification according to the USDA system. In order to, derive the input parameters required by the STICS model, several pedo-transfer functions were applied, following the methodology described by Brisson et al. [15]. These functions allowed the estimation of hydraulic and physical soil properties, such as field capacity, wilting point, and bulk density. Additionally, terrain data, including elevation, slope, and aspect, were extracted from the GTOPO30 high-resolution digital elevation model (DEM) [62] to account for spatial heterogeneity in topography, which influences microclimatic conditions and vine performance.

### 4.5. Modelling Assumptions

To enable regional-scale simulations, a set of standard modelling assumptions was adopted to ensure consistency across the study area. Touriga-Franca, the most widely planted variety in the Douro, was used as a single representative grapevine variety, and was simulated across all grid points based on the model calibration conducted by Fraga et al. [19], which is appropriate for the predominant cultivars of the Douro region. For the previous calibration study in the Douro region [19], the STICS crop model showed strong performance in simulating grape harvest dates and yield for the target variety, Touriga-Franca. Harvest timing was predicted with high accuracy, deviating by only 1 day on average and showing strong statistical agreement (R^2^ = 0.69, MAE = 0.65). Yield estimates were also reliable, with high model efficiency (MAE = 0.81, R^2^ = 0.86). Alcohol content showed a MAE of 1% vol. Additionally, STICS was also effective in simulating other variables, such as varietal differences in vine water stress (R^2^ up to 0.94).

Herein, crop management was also assumed to be homogeneous throughout the Douro region in order to obtain spatial assessment. A uniform plant density of 3000 plants per hectare was assumed throughout, reflecting common regional viticultural practices. The training system was defined a bilateral cordon, a widely used trellis structure in the region. These assumptions allowed for the spatialization of results while maintaining biological and agronomic relevance across the heterogeneous landscape of the Douro Valley.

### 4.6. Model Simulations

The STICS crop model was driven with daily climate, soil, terrain, and vineyard management data to perform spatially explicit simulations across the Douro wine region. Simulations were conducted independently for each combination of GCM and climate scenario (SSP1–2.6 and SSP5–8.5), allowing the assessment of both individual model outputs and ensemble statistics. The 4-member ensemble median was calculated to represent the central tendency of projected outcomes, while ensemble uncertainty was quantified using the interquartile range (IQR) of the ensemble. Key viticultural variables simulated included annual grape yield (t/ha), estimated alcohol content (% vol), and flowering date (Julian Day). These variables were computed for both the historical baseline period (1986–2015) and the future period (2041–2070). Differences between the historical and future periods were analysed to evaluate potential climate-driven changes in viticultural performance under contrasting emissions scenarios. An illustration of the model runs is shown in Figure 2.

## 5. Conclusions

Climate change is projected to substantially alter viticultural conditions in the DWR, with significant and spatially variable impacts on grapevine yield, flowering timing, and potential alcohol content, under both emission scenarios. These changes highlight the critical need for developing localized adaptation strategies that reflect the specific environmental and production conditions of the DWR. Given the projected variability in climate impacts across sub-regions, adaptation must be grounded in fine-scale assessments and supported by robust scientific evidence. Equally important is the active involvement of stakeholders throughout the process, ensuring that producers, institutions, and policymakers are aligned in interpreting climate risks and identifying feasible responses. Strengthening communication between researchers and practitioners, promoting capacity-building, and fostering participatory planning will be essential to translate complex projections into meaningful action. The long-term resilience of Douro viticulture will depend not only on technical innovation but also on shared understanding, coordinated adaptation, and sustained engagement across the wine sector.

## Figures and Tables

**Table 1:** Description of the General Circulation Models (GCMs) used in this study.

Model	Institution	Country	Original Resolution	Reference
CanESM5	Canadian Centre for Climate Modelling and Analysis	Canada	250 km	[57]
EC-Earth3	EC-Earth Consortium	Europe	80 km	[58]
IPSL-CM6A-LR	Institut Pierre-Simon Laplace	France	160 km	[59]
MPI-ESM1-2-HR	Max Planck Institute for Meteorology	Germany	80 km	[60]

**Table 2:** Soil and terrain datasets and references used to obtain the necessary parameters of the STICS crop model. Parameters used in pedo-transfer functions are highlighted by *.

Parameter	STICS Parameter	Dataset/Calculation
Albedo of the dry soil	albedo	[63]
Clay content in the surface layer (%)	Argi	HWSD
Cumulative evaporation limit (mm)	q0	[64]
Dominant soil unit (FAO)	*	HWSD
Elevation (m)	*	GTOPO30
Fraction of runoff in soil	ruisolnu	[65]
Limestone content in the surface (%)	Calc	HWSD
Orientation	*	GTOPO30
Permeability classes	*	[66]
Slope (%)	*	GTOPO30
Soil calcium carbonate content	*	HWSD
Soil depth	*	HWSD
Soil pH	pH	HWSD
Texture class	*	HWSD
USDA Texture	*	HWSD

## Data Availability

All data will be made available upon reasonable request to the corresponding author(s).

## Abbreviations

The following abbreviations are used in this manuscript:

DWR	Douro wine region
